# Noise-Driven Causal Inference in Biomolecular Networks

**DOI:** 10.1371/journal.pone.0125777

**Published:** 2015-06-01

**Authors:** Robert J. Prill, Robert Vogel, Guillermo A. Cecchi, Grégoire Altan-Bonnet, Gustavo Stolovitzky

**Affiliations:** 1 IBM T. J. Watson Research Center, 1101 Kitchawan Road, Route 134, Yorktown Heights, N.Y. 10598, United States of America; 2 ImmunoDynamics Group, Program in Computational Biology and Immunology, Memorial Sloan- Kettering Cancer Center, 1275 York Avenue, Box 460, New York, N.Y. 10065, United States of America; Leibniz-Institute for Farm Animal Biology (FBN), GERMANY

## Abstract

Single-cell RNA and protein concentrations dynamically fluctuate because of stochastic ("noisy") regulation. Consequently, biological signaling and genetic networks not only translate stimuli with functional response but also random fluctuations. Intuitively, this feature manifests as the accumulation of fluctuations from the network source to the target. Taking advantage of the fact that noise propagates directionally, we developed a method for causation prediction that does not require time-lagged observations and therefore can be applied to data generated by destructive assays such as immunohistochemistry. Our method for causation prediction, "Inference of Network Directionality Using Covariance Elements (INDUCE)," exploits the theoretical relationship between a change in the strength of a causal interaction and the associated changes in the single cell measured entries of the covariance matrix of protein concentrations. We validated our method for causation prediction in two experimental systems where causation is well established: in an *E. coli* synthetic gene network, and in MEK to ERK signaling in mammalian cells. We report the first analysis of covariance elements documenting noise propagation from a kinase to a phosphorylated substrate in an endogenous mammalian signaling network.

## Introduction

Correlation does not imply causation. True in general, the adage relents under technical definitions of causation. For example, a time series *X* is Granger-causal of a time series *Y* if future values of *Y* can be significantly predicted from current and past values of *X* using a series of t- and F-tests on regressions [1]. Non-temporal arguments can also support a type of causal inference. A “surgical” intervention, according to Pearl, is the act of replacing one of the variables in a system by a constant value [2]. Comparing pre- and post-intervention distributions can support causal inference—e.g., *X* is topologically upstream of *Y*. In a Bayesian network, causality between variables represented by nodes is modeled as a directed acyclic graph—i.e., feedbacks are prohibited [3]–that represent the structure of conditional dependences between the nodes. Despite the appeal of these different approaches to determine causality, all causal inference methods pose some difficulties. A network with nodes interacting with vastly different time scales can pose problems for causal interpretation by methods evoking time lag analysis that cannot bridge the range of time scales at play in the system. A model that by design does not incorporate feedback loops, would likely fail to approximate reality when feedbacks indeed exist and play a role in the causal relationships.

The inference of causal interactions in networks is of particular importance in the context of biological systems. Individual cells utilize networks of interacting molecules to process environmental stimuli and execute a response. To understand these systems such that we can effectively control cellular behavior, it is necessary to deduce the causal relationships among the set of interacting biomolecular species. Dunlop *et al.* [4] demonstrated a form of causal inference of gene interactions by measuring temporal correlations of fluorescent proteins embedded in an *E. coli* synthetic gene regulatory network. The utility of the correlation function in this context is due to the stochasticity, “noise”, of gene expression [5] and the transmission of stochastic fluctuations within a network [6]. Such reliance on stochastic variability to infer causality in biological networks is in fact generically applicable. Noise in gene expression is not limited to *E. coli* synthetic networks [7], as single cell studies have demonstrated that proteins are expressed in exponentially distributed bursts resulting in long tailed distributions of protein at the steady state [8, 9]. Furthermore, long tailed distributions of gene products have been shown to be phylogenetically universal [7, 8]. As a result, noise based causal inference techniques may be applicable to many biological systems.

In contrast to gene regulatory networks studied in *E. coli* and *S. cerevisiae*, stochasticity in the signaling networks of mammalian cells is ill-understood. Signaling proteins can be expressed at thousands of copies per mammalian cell [10, 11], and therefore the substantial diversity in signaling protein concentration that has been observed [12] is clearly not attributable to low-copy-number mechanisms as may be the case for *E. coli*. It is possible that signaling networks propagate stochastic fluctuations in phosphoprotein activities analogous to what has been observed in *E. coli* transcription, but this has yet to be demonstrated experimentally. However, the recent progress of single cell immunohistochemical based technologies, e.g. flow cytometry, fluorescent imaging, and mass cytometry, provide robust ensemble measurements of protein phosphorylation states. These techniques are destructive assays, which preclude a temporal tracking of phosphorylation states in single cells. Causal reconstructions of signaling networks have mainly relied on integrating perturbations (interventions in the language of Pearl [2]), and analyzing the results using a Bayesian network model [13, 14] or other statistical tools (conditional probability and density estimate [15]).

Here we introduce a new causal inference method that analyzes the structure of the covariance matrix between putatively causally related molecular species without following these species in time or requiring surgical interventions *à la* Pearl. Our method, which we call INDUCE (Inference of Network Directionality Using Covariance Elements), is based on a stochastic model that integrates experimental perturbations of the network connectivity. Similar to previous work characterizing biochemical stochasticity [16–19], our causality prediction is grounded in the formalism of stochastic differential equations. We validated the method in two experimental systems where ground truth causation is known, an *E. coli* synthetic regulatory gene network [6] and MAPK signaling in T cells. While there has been several studies documenting the transmission of fluctuations through mammalian signaling networks [20–22], our study uniquely defines and experimentally validates functional relationships between elements of the covariance matrix of signaling components. We do not attempt to derive precise numerical values for the parameters of the theory, thus in this sense our study is qualitative. Overall, our study provides a novel framework for deducing biochemical interactions by analyzing the distribution of phospho-protein in single cell measurements.

## Results

### Theory

Our premise is that fluctuations of network elements is related to their connectivity. Therefore, variances and covariances of the variables associated to the interconnected elements may be used to infer some of the network connectivity structure. To determine the functional relationship of covariance elements, consider a generic linear stationary stochastic dynamical system represented by the stochastic differential equation

$$\begin{array}{c} \frac{dx_{j}\left(t\right)}{dt}=\sum_{i=1}^{N}a_{ji}x_{i}\left(t\right)+q_{ji}\xi_{i}\left(t\right), \end{array}$$ (1)

where *x*_*i*_(*t*) is the deviation of the *i*-th variable from its average, *ξ*_*i*_(*t*) is a white noise independent of *ξ*_*j*_(*t*) (*i* ≠ *j*), *a*_*ji*_ *x*_*i*_ is the rate of change of variable *j* due to the presence of variable *i*, and *q*_*ji*_ is the magnitude of the *i*-th white noise acting on the *j*-th variable. Eq 1 is a multivariate linear Langevin equation, which in this case is describing the dynamics of a system of chemical reactions driven by random fluctuations of scale *q* about its steady state. Historically, the Langevin equation was used in the statistical description of Brownian motion; it was much later that it was adapted to chemical systems as a first order approximation to the chemical master equation [16–19, 23]. The delta-correlated white noise terms *ξ*_*i*_(*t*) are defined as

$$\begin{array}{ccc} \left\langle \xi_{i}\left(t\right)\right\rangle & = & 0\\ \left\langle \xi_{i}\left(t\right)\xi_{j}\left(t^{\prime }\right)\right\rangle & = & \delta_{ij}\delta \left(t-t^{\prime }\right), \end{array}$$ (2)

where brackets denote averaging over possible temporal realization of the white noise, *δ*_*ij*_ is the Kronecker delta, and *δ*(*t*) is Dirac’s delta function. Written in matrix notation, Eq 1 takes the simpler form

$$\begin{array}{c} \frac{dx(t)}{dt}=Ax\left(t\right)+Q\xi \left(t\right), \end{array}$$ (3)

where **x**(*t*) is an *N* dimension column vector (dimension *N* × 1), **A** is the *N* × *N* matrix that determines the connectivity of the network of interaction between the variables *x*_*i*_, and **Q** is an *N* × *M* matrix which represents the strength of the *M*-dimensional noise column vector **ξ**(*t*) on the rate of change of the dynamic variables. A formal solution to Eq 3 can be written as

$$\begin{array}{c} x\left(t\right)=\int_{-\infty }^{t}e^{A(t-s)}Q\xi \left(s\right)\;ds. \end{array}$$ (4)

For our linearized system, the network inference problem is the problem of reconstructing **A** from **x**(*t*). From Eq 4, it would seem that solving for **A** requires knowing **Q**, which is usually unknown. Fortunately, the inference problem can be recast such that **Q** is absorbed into terms that are observable so that **Q** need not be known or estimated. Using Eq 4 we can write the lagged covariance matrix of **x**(*t*)

$$\begin{array}{c} \Sigma \left(\tau \right)=\langle x\left(t\right)x^{T}\left(t+\tau \right)\rangle =\int_{-\infty }^{t}e^{A(t-s)}QQ^{T}e^{A^{T}\left(t-s\right)}e^{A^{T}\tau }\;ds \end{array}$$ (5)

and taking e^**A**^*T*^*τ*^ outside of the integral we obtain

$$\begin{array}{c} \Sigma \left(\tau \right)=\Sigma_{o}\;e^{A^{T}\tau }, \end{array}$$ (6)

where **Σ**_*o*_ ≡ **Σ**(*τ* = 0) is the (unlagged) covariance matrix, which could in principle be computed from the data. Eq 6 specifies the theoretical relationship between the lagged covariance matrix **Σ**(*τ*), the covariance matrix **Σ**_*o*_, and the network matrix **A**. Note that the noise-strength matrix **Q** is absorbed into the unlagged covariance matrix. Eq 6 suggests a possible strategy for network inference, which can be used when the multivariate time-series **x**(*t*) is observed with adequate temporal resolution with respect to the time constants of the linear system, contained in the spectrum of the connectivity matrix **A**. Eq 6 is consistent with the common notion that temporal observations enable causal inference because cause precedes effect. However, Eq 6 cannot be used when the time courses of the molecular entities of interest cannot be measured, or when the measurements do not allow for a time resolution faster than the fastest time scale of the system.

Next we explore the possibility of extracting information of the connectivity matrix **A** from the covariance matrix without temporal information. The stochastic dynamic equation, Eq 1, was developed as a stationary process and therefore its covariance matrix is time invariant. Therefore, taking the derivative with respect to *t* in both members of Eq 5 and using that $\frac{d}{dt}\text{Σ}\;(\tau )=0$ we find that

$$\begin{array}{c} A\Sigma \left(\tau \right)+\Sigma \left(\tau \right)A^{T}+QQ^{T}e^{A^{T}\tau }=0. \end{array}$$ (7)

We are interested in the unlagged covariance matrix, for which we set *τ* = 0. Therefore from Eq 7 it follows that

$$\begin{array}{c} \begin{array}{c} A\Sigma_{o}+\Sigma_{o}A^{T}+QQ^{T}=0. \end{array} \end{array}$$ (8)

Eq 8, a version of the Lyapunov equation [17], specifies the theoretical relationship between the network matrix **A**, the (unlagged) covariance matrix **Σ**_*o*_, and the matrix **Q**. Eq 8 will form the basis of our network inference strategy. Assuming for the moment that **Q** **Q** ^*T*^ is known (it is not known in general), there are *N* ^2^ unknowns in the matrix **A** but only *N*(*N*+1)/2 equations in Eq 8, considering the symmetry in the equations. This is, in our restricted case, the reason why correlation does not imply causation: the covariance matrix **Σ**_*o*_ is consistent with an infinite number of choices for **A**. Indeed, a general solution for Eq 8 can be written as:

$$A=(-\frac{1}{2}QQ^{T}+U)\Sigma_{0}^{-1},$$ (9)

where **U** is an undefined antisymmetric matrix. Therefore Eq 8 restricts the solution **A** up to the *N*(*N*−1)/2 parameters needed to determine **U**. When **A** is known to be symmetric Eq 8 is sufficient to determine **A**. **A** is symmetric, for example, when we are considering the dynamics of a conservative physical system around its equilibrium point. In such case **A** is the Hessian of the potential energy of the system at the assumed fixed point. Eq 8 can be easily solved if besides the symmetry of **A**, we assume that the noise terms represent a thermal bath of a physical system. In that case **Q** **Q** ^*T*^ is an isotropic matrix, that is **Q** **Q** ^*T*^ = *β* ^−1^ **I**, where *β* = 1/*k*_*B*_ *T*, *k*_*B*_ is the Boltzmann constant, *T* is the bath temperature and **I** is the identity matrix. In this case

$$\begin{array}{c} A=-\Sigma_{o}^{-1}k_{B}T \end{array}$$ (10)

From the point of network reconstruction Eq 10 is a curious result, as the intuition suggests that the strength of the connection between *i* and *j* is proportional to the correlation between *i* and *j*. However, Eq 10 clearly shows that an *i*-*j* connection is proportional to the *ij* element of the inverse of the covariance matrix.

In summary, temporal measurements of **x**(*t*) can be used to calculate the lagged covariance matrix **Σ**(*τ*), enabling a reconstruction of a non-symmetric network matrix **A** via Eq 6. If time lagged experiments are not possible, **x**(*t*) can be used to calculate the unlagged covariance matrix **Σ**_*o*_, which however doesn’t have enough information to determine **A** via Eq 8, unless we have reasons to assume that **A** is symmetric. Even when **A** is not symmetric, we can still use Eq 9 and choose **U** by optimization techniques under the constraint that **A** is maximally sparse. We will not pursue this heuristic idea in this paper.

The original contribution in what follows is the adaption of Eq 8 for causal inference—i.e., to infer a non-symmetric network matrix **A**—using our novel inference method we call INDUCE.

### INDUCE: Inference of Network Directionality Using Covariance Elements

The key insight of our inference method is that the covariance matrix changes in a theoretically predictable way (specified by Eq 8) in response to changes in strength of the network connectivity caused by external perturbations. A simple example illustrates the basic idea behind INDUCE. Consider a network composed of single directional edge spanning two nodes, and a diagonal noise matrix,

$$\begin{array}{c} A=[\begin{array}{cc} -\lambda_{1} & 0\\ k & -\lambda_{2} \end{array}],\;Q=[\begin{array}{cc} q_{1} & 0\\ 0 & q_{2} \end{array}]. \end{array}$$

Setting *λ*_1_ = *λ*_2_ = *λ* to improve clarity and solving Eq 8 for **Σ**_*o*_ gives

$$\begin{array}{c} \Sigma_{o}=[\begin{array}{cc} \alpha & \frac{\alpha k}{2\lambda }\\ \frac{\alpha k}{2\lambda } & \beta +\frac{\alpha k^{2}}{2\lambda^{2}} \end{array}] \end{array}$$ (11)

where $\alpha =\frac{q_{1}^{2}}{2\lambda }$ and $\beta =\frac{q_{2}^{2}}{2\lambda }$ are components of the variances that do not depend on the network connectivity.

Next, we analyze how **Σ**_*o*_ depends on the strength of the interaction *k*. Let’s denote the covariance between *x*_1_ and *x*_2_ as *σ*_12_ and the respective variances as $\sigma_{i}^{2}$. When *k* = 0 (i.e., the nodes are disconnected), *σ*_12_ = 0 and $\sigma_{2}^{2}=\beta$. For *k* ≠ 0, $\sigma_{2}^{2}$ is proportional to *k* ^2^ whereas $\sigma_{1}^{2}$ does not depend on *k*. Of particular importance for causal inference, $\sigma_{1}^{2}$ and $\sigma_{2}^{2}$ are differentially sensitive to changes in *k*. Taking advantage of this theoretical result, a strategy for causal inference is to observe **Σ**_*o*_(*k*) for a collection of networks that differ only in the magnitude of the connection strengths (parameterized by *k*). This strategy might be exploited in a number of real-world systems in which the connection strengths can be controlled externally. To implement this strategy, we only require a means of changing *k*; knowing the actual value of *k* is not required. Rearranging the previous result we have

$$\begin{array}{c} \Sigma_{o}=[\begin{array}{cc} \alpha & \sigma_{12}\\ \sigma_{12} & \beta +2\sigma_{12}^{2}/\alpha \end{array}], \end{array}$$ (12)

the theoretical covariance matrix expressed in terms of *σ*_12_ for a network composed of a single directed edge spanning two nodes. For this connectivity, Eq 8 predicts that $\sigma_{2}^{2}$ is a quadratic function of *σ*_12_ whereas $\sigma_{1}^{2}$ does not depend on *σ*_12_. This analysis can be performed for any hypothetical network connectivity, though beyond three to four nodes, the analytical expressions for **Σ**_*o*_ are cumbersome.

Note that this theory applies to a single cell observed over time where the sampling of **x**(*t*) enables estimating **Σ**_*o*_. To apply this theory to situations in which protein concentrations in single cells are not followed over time but rather are measured from many clonal cells under the same conditions (e.g., as done in flow cytometry, mass cytometry, and fluorescence imaging) we need to evoke the assumption of ergodicity. This assumption postulates that the statistics resulting from sampling many identical cells at one time-point is equivalent to the statistics resulting from sampling one cell at many time-points during a stationary phase. The ergodic assumption enables the estimation of **Σ**_*o*_ from a population of identical cells (e.g., genetic clones). This assumption is commonly evoked when modeling and analyzing biochemical noise [6].

### Biomolecular networks

In several cellular processes the response caused by the presence of specific molecules, such as rate of transcription of a gene as a function of the concentration of its transcription factor, or the rate of phosphorylation of a protein as a function of the concentration of its kinase, is typically a nonlinear, saturating function of the concentration of the causative molecule. It is customary to model the response *f*_*ji*_ of molecule *j* in terms of the concentration *x*_*i*_ of a causative molecule *i* (e.g., the transcription factor or the kinase), using a Hill equation

$$\begin{array}{c} f_{ji}\left(x_{i};\vec{\theta_{ji}}\right)=\nu_{ji}\frac{x_{i}^{n_{ji}}}{K_{ji}^{n_{ji}}+x_{i}^{n_{ji}}},\;\;\vec{\theta_{ji}}=\left\{\nu_{ji},n_{ji},K_{ji}\right\} \end{array}$$ (13)

where $\vec{\theta_{ji}}$ represents the parameters of the response function, in this case *ν*_*ji*_ (the saturated size of the response), *K*_*ji*_ (the concentration of *x*_*i*_ producing half the response), and *n*_*ji*_ (the Hill coefficient modeling a source of biochemical nonlinearity such as cooperativity between the causative molecules). In general, the strength of the biochemical interaction between the response molecule and the causative molecule can in many cases be experimentally manipulated using external perturbations such as receptor ligands, or intracellular drugs and toxicants.

**Fig 1:**
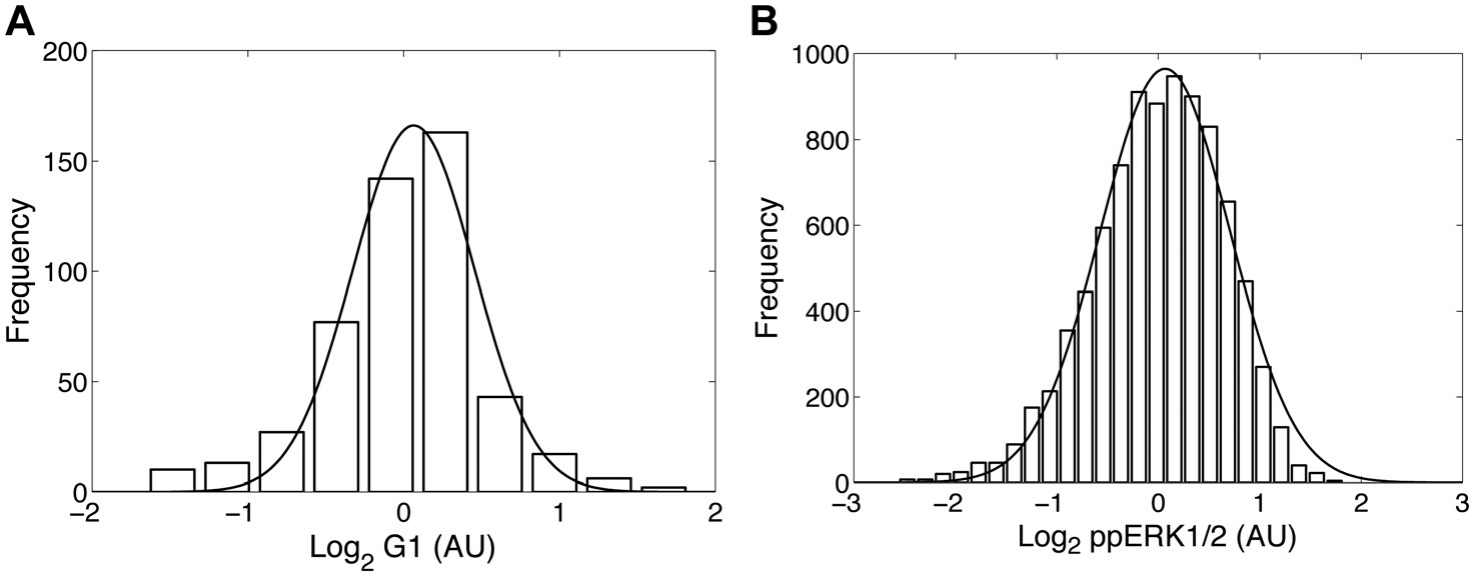
Biological noise is well approximated by the lognormal distribution. (A) Single-cell quantification of fluorescent CFP in *E. coli* treated with 2 mM IPTG. (B) Flow cytometry measurements of ERK1/2 phosphorylated at (T202, Y204) in mouse T cells treated with 50nM PMA.

It is customary to approximate the stochastic dynamics of chemical products in chemical reactions by the chemical Langevin equation (CLE), which incorporates the fluctuations arising from the small number of discrete interacting chemical species [23]. While an attractive starting point, this approach fails to describe two important experimental observations, namely the often observed lognormal nature of the distribution of biochemical reaction products, and the observed non-vanishing fluctuations even for large numbers of molecules (Fig 1 and Fig. A in S1 text). To remedy these insufficiencies we extended the CLE formalism in two ways. First, we account for the logarithmic fluctuations of biochemical products by modeling the dynamics of the logarithm of the concentration for each species. In this case, Eq 13 can model the response function in log-coordinates. Secondly, we account for the stochasticity originating from fluctuations in biochemical reaction rate parameters. (The complete derivation is provided in Section B in S1 text.) After invoking these considerations and linearization we derive a stochastic differential equation equivalent to Eq 3.

$$\begin{array}{cc} \frac{d\delta y_{j}}{dt} & =\sum_{i\neq j}(a_{ji}\delta y_{i})-\langle \lambda_{j}\rangle \delta y_{j}+q_{j}\epsilon_{j} \end{array}$$ (14)

Where *δy*_*j*_ is the logarithmic deviation from the mean of biochemical species *j*, $a_{ji}=\langle \lambda_{j}\rangle \partial \ln(f_{ji})/\partial \ln(x_{i}){|}_{x_{i}=\langle x_{i}\rangle }$ is the logarithmic rate of production of species *j* dependent on species *i*, *λ*_*j*_ is the biochemical decay constant of species *j*, *q*_*j*_ is the magnitude of the intrinsic logarithmic fluctuations of species *j*, and *ϵ*_*j*_ is a white noise random variable. The equivalence of the above equation with Eq 3 permits the utilization of the INDUCE analysis for the causal inference in biomolecular networks.

**Fig 2:**
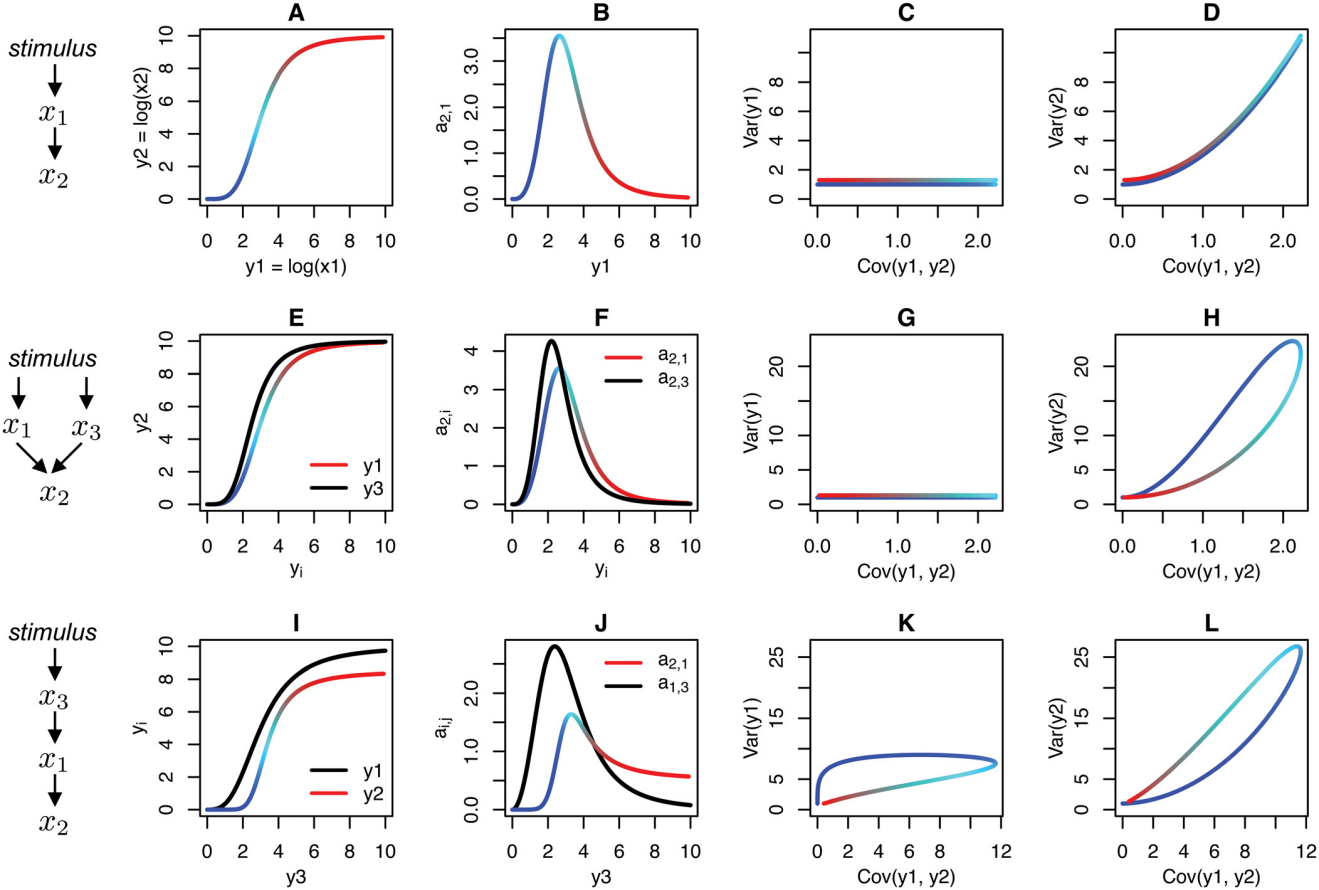
INDUCE (Inference of Network Directionality Using Covariance Elements) analysis for hypothetical model networks. (A-D) INDUCE analysis of an isolated network connection. From left to right, (A) the Hill equation model of log concentration transfer function is color registered to *y*_2_. (B) The derivative of the transfer function is the network connection strength *a*_2,1_, which peaks at half-maximal activation. (C-D) Variance versus covariance plots, the solution to Eq 8 applied at closely-spaced fixed points along the transfer function shown in A. (E-H) INDUCE analysis of a convergent (additive) regulation of node 2. The transfer function *y*_2_ versus *y*_3_ shown in black. (F) The connection strength *a*_2,3_ shown in black. (H) Var(y2) versus Cov(y1, y2) has a loop, a hallmark of differential sensitivities to a stimulus. (I-L) INDUCE analysis of a linear cascade. (K, L) Both variance versus covariance plots exhibit loops, a hallmark of differential sensitivities to a stimulus. In all examples, biochemical parameters were chosen to illustrate qualitative differences in the variance versus covariance plots for different connectivities (see Section D in S1 text for parameter values).

We applied the INDUCE analysis to small network motifs intended to represent plausible connectivities linking a pair of measured proteins. Our approach is represented graphically in Fig 2, where we show the dependence of the covariance matrix elements to changes in connectivity strength induced by a dose-response experiment (i.e., sampling along the response function shown in Fig 2A).

First, we studied the interdependence of the covariance matrix elements for a single source-target protein pair (Fig 2A). We plot the values of the covariance matrix entries predicted by Eq 11, the solution of Eq 8 for a single network connection and diagonal noise matrix, where the reaction rate (*k* in Eq 11) is the instantaneous slope of the response function, shown in Fig 2B. The effect of the parameter *k* (renamed *a*_2,1_ in the figure to remove ambiguity in larger networks) is explored in the parametric plots of the entries of the covariance matrix (Fig 2C and 2D). As indicated in Eq 12, the variance of the deviations of the *source* protein ($\sigma_{1}^{2}$) does not depend on the covariance (*σ*_12_) whereas the variance in the deviations of the *target* protein ($\sigma_{2}^{2}$) is a quadratic function of *σ*_12_.

Next, we analyzed a network in which protein 2 is co-regulated by proteins 1 and 3 additively (Fig 2E and 2H). As might be anticipated, the *source* variance ($\sigma_{1}^{2}$) is unaffected by downstream changes to the network connectivity. However, the effect of dual-regulation of protein 2 on $\sigma_{2}^{2}$ is more complex, in that $\sigma_{2}^{2}$ is now a quadratic with respect to *σ*_12_ and *σ*_32_ (Equation S15c in S1 text). Differences in the sensitivity of protein 1 and protein 3 to the stimulus will cause $\sigma_{2}^{2}$ to change values independent to *σ*_12_, manifesting as a deviation from the two node covariance path. The absolute magnitude of the deviation from the two node predictions is dependent on the biomolecular system parameters. It is intriguing that an analysis of the dependence of the covariance matrix elements performed for a pair of interacting proteins might be used to infer the existence and connectivity of a third *unmeasured* protein.

We also analyzed a linear cascade in which protein 3 regulates protein 1, and protein 1 regulates protein 2 (Fig 2I and 2L). The complex path of $\sigma_{1}^{2}$ and $\sigma_{2}^{2}$ is unique of the INDUCE analysis of noise propagation from a common source with disparate sensitivity to the stimulus. Some of the fluctuations in protein 3 propagate to protein 2 via protein 1. As in the case of the previous example, the extent of the deviation from the two-node covariance path depends on the particular biomolecular parameterization. It is intriguing that the INDUCE method might be used to infer the existence and connectivity of previously unknown and *unmeasured* protein, using the complexity of $\sigma_{1}^{2}$ and $\sigma_{2}^{2}$ as a indicator that an unknown and unmeasured source of fluctuations is upstream of proteins 1 and 2.

“Open loop” trajectories in the variance-covariance plot (such as those in Fig 2H, 2K, and 2L) do not occur for all parameterizations of the three node network motifs (see Fig. B in S1 text for additional parameterizations). The presence of an open-loop trajectory in the variance-covariance plot is sufficient to implicate an unmeasured noise source. The absence of an open loop trajectory, however does not discount the existence of an unmeasured node. The theory and modeling experiments clarify that multiple noise sources have the potential to interact, or not, in the generation of the covariance matrix of the measured nodes, depending on the biomolecular parameterization. Of practical utility, it may be possible to treat a network connection in a larger network as if it is an isolated network connection from the perspective of noise propagation when the interaction of multiple noise sources is minimal.

Similar analysis can be performed for other connectivities. These results show that an analysis of the interdependences of the covariance matrix elements can in principle discriminate the directionality of information transfer between two nodes, even in the context of additional unmeasured protein affecting the source or the target proteins.

### E. coli gene expression

We analyzed a published gene expression dataset generated with an *E. coli* plasmid encoding a three gene synthetic transcriptional network. The parent gene in the network (G0) encodes the lac repressor and is constitutively transcribed. Unless exogenous IPTG (a lactose analog) is present, the lac repressor inhibits the transcription of gene 1 (G1). G1 bicistronically transcribes *tetR* (tetracycline repressor) and *cfp* (Cyan Fluorescent Protein) which serve two functions, first a repressor of gene 2 (G2) transcription and second a measure of G1 activity. G2 exclusively encodes for the Yellow Fluorescent Protein (YFP), providing a measurable quantity of G2 activity [6]. This simple genetic circuit was implemented as a synthetic experimental network and therefore the ground truth connectivity underlying the measured concentrations was known.

**Fig 3:**
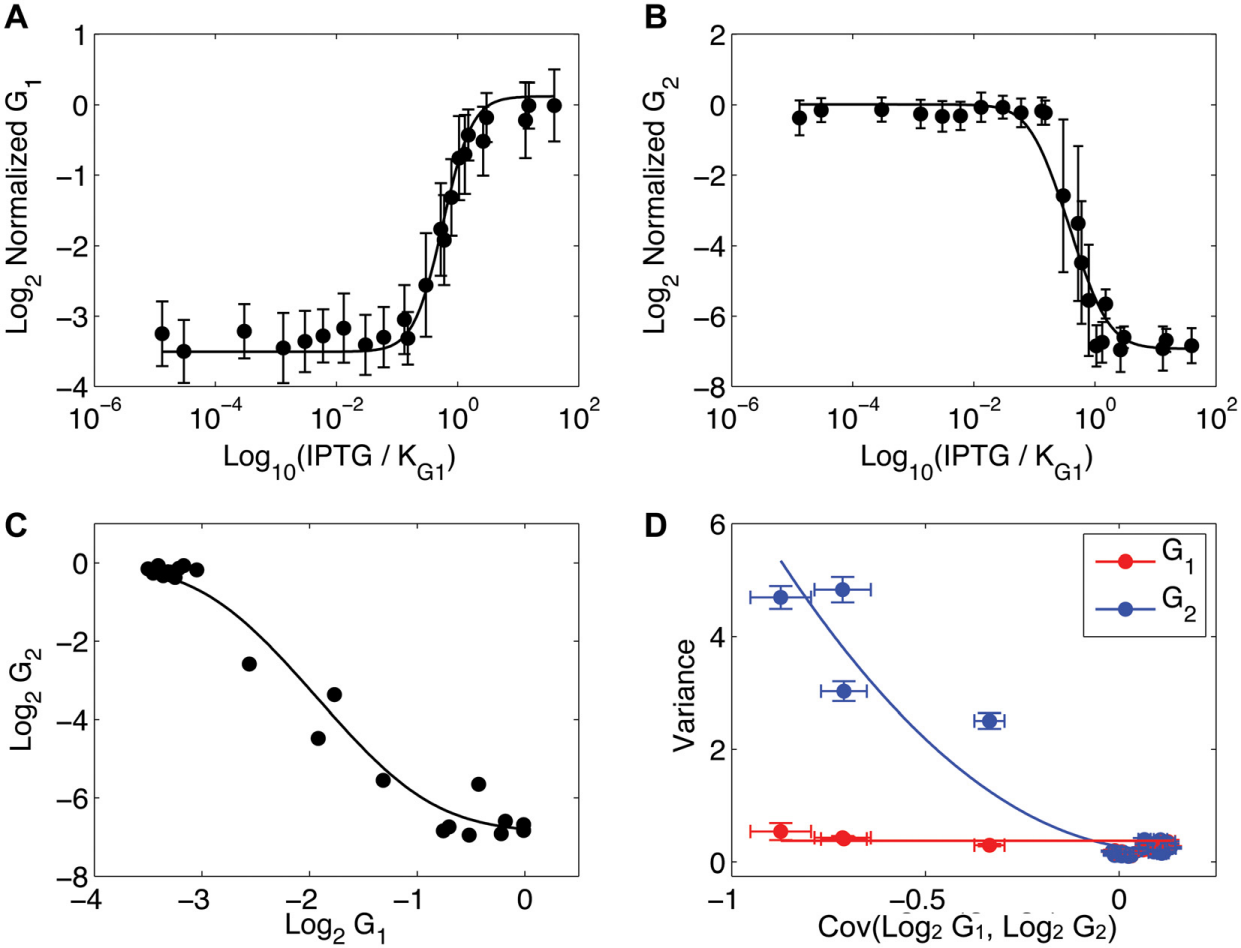
*E. coli* synthetic transcriptional network. (A) IPTG dose-response of G1. (B) IPTG dose-response of G2. (A-B) Averages of the log of the normalized G1 (A) and G2 (B) concentrations ±1 s.d. (C) Transfer function of G1 versus G2 (log scale). The negative slope is indicative of the repression of G2 by G1. (D) INDUCE analysis showing the fit of a two-node network model (solid curves). Error bars are 1 s.d. in computed in 1000 bootstraps of the data.

Our analysis of *E. coli* gene expression is shown in Fig 3. Fig 3A and 3B are the dose-responses of G1 and G2 as a function of IPTG concentration. G1 is a repressor of G2 transcription. This repressive functional relationship is clearly seen from the decreasing sigmoidal function fitted to the average concentrations of log G1 versus log G2 (panel C). The slope of the transfer function is proportional to the strength of the regulatory effect of G1 on G2. It is crucial to realize that neither the dose-responses nor the transfer function contain any signal supporting a causal inference.

In the transfer function plot (Fig 3C), most IPTG doses are clustered at the high or low saturation points. Four IPTG doses are the in the most rapidly changing region of the transfer function. The transfer function plot indicates that five different network connection strengths were observed for the *E. coli* network, zero connectivity at saturation, and four intermediate connection strengths in the dynamic range of the transfer function.

Fig 3D shows the INDUCE analysis using a two-node network model (see Fig 2B). As predicted by the model, the variance of G1 does not depend on the covariance between G1 and G2, Cov(G1, G2), whereas the variance of G2 is well fitted by a quadratic function of Cov(G1, G2). Interpreted through the lens of the INDUCE analysis, the data predicts that G1 regulates G2, as is indeed the case in this system. That the regulation is a repression can be inferred from the negative sign of Cov(G1, G2).

A criticism of the model fit is that a denser sampling in the dynamic range of the transfer function would be desirable to more thoroughly trace out the covariance matrix sensitivity to a changing connectivity strength. Since this is a re-analysis of a historical data set, this was not possible. In the next validation experiment, we were able to sample the dynamic region of the transfer function more thoroughly using our own experimental setup. (Details of the data normalization and fitting procedures are provided in Section F in S1 text.)

### MAP kinase signaling

MEK and ERK participate in a well-characterized phosphorelay signaling system within the MAP kinase pathway [24]. We measured the concentrations of the phosphoproteins MEK-pS (pMEK) and ERK-pTpY (ppERK) in mouse T cells using immunostaining and multiparameter flow cytometry as previously described [12]. We stimulated the T cells with phorbol 12-myristate 13-acetate (PMA), a chemical activator of protein kinase C (PKC), part of the canonical MAP kinase pathway, PKC → Ras → Raf → MEK → ERK. Then, we measured the concentrations of pMEK and ppERK ten minutes after PMA stimulation. The PMA stimulus is specific in the sense that pre-treatment with a MEK inhibitor completely blocks ERK phosphorylation. Using flow cytometry we quantified the intensity of fluorescent dyes bound to antibodies directed against the phosphoproteins pMEK and ppERK in individual mouse T cells. Previously, we verified that fluorescence intensity in our experimental protocol scales linearly with protein concentration over many decades of dynamic range [12]. The data can be downloaded [25, 26].

**Fig 4:**
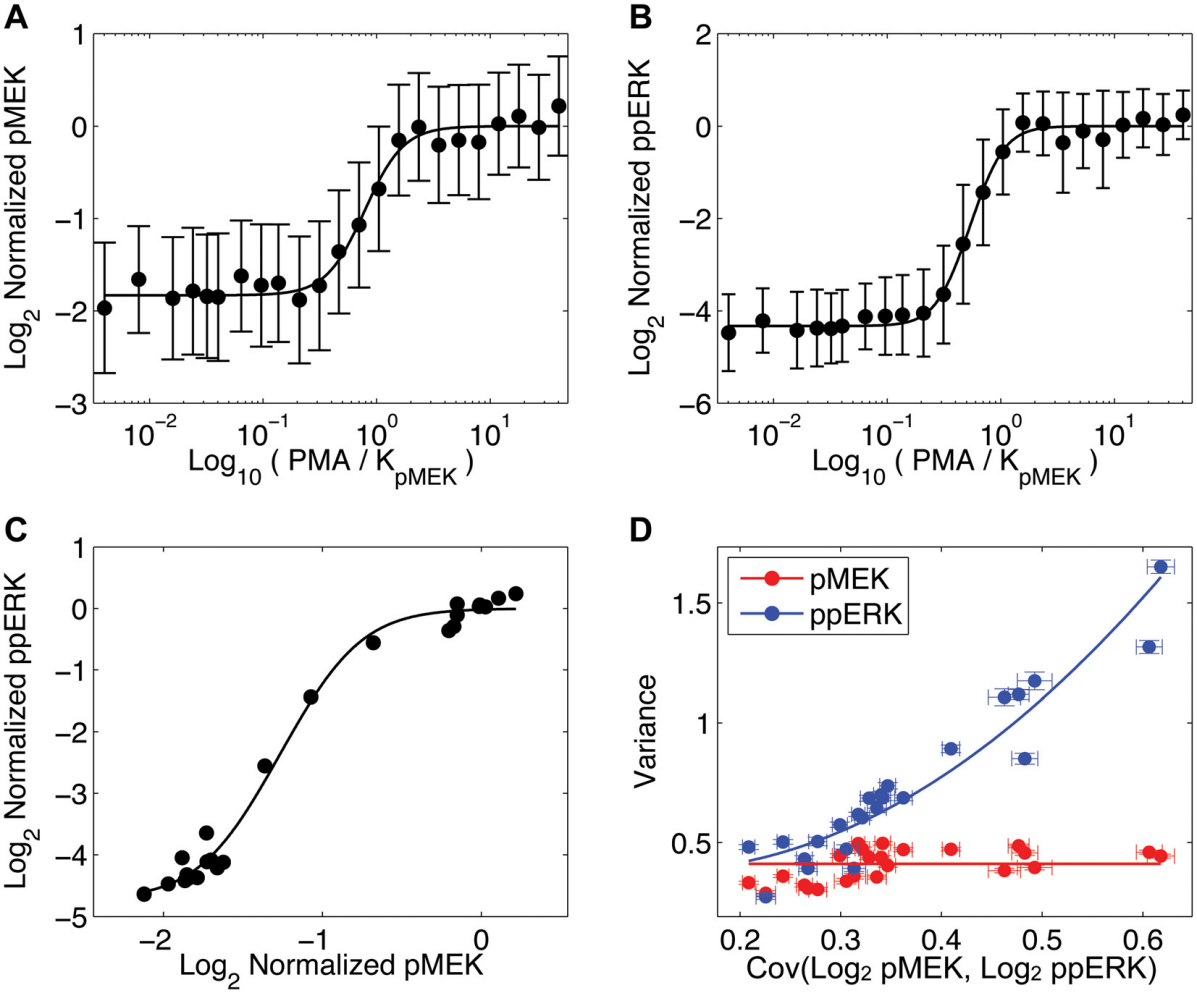
MAP kinase signaling. (A) PMA dose-response of pMEK. (B) PMA dose-response of ppERK. (A-B) Averages of the log of the normalized protein concentrations ±1 s.d. (C) Transfer function of pMEK versus ppERK. The positive slope is indicative of ERK activation by MEK. (D) INDUCE analysis showing the fit of two-node network model (solid curves). Error bars show 1 s.d. in 1000 bootstraps of the data.

Our analysis of the MEK-ERK phosphorelay is shown in Fig 4. Fig 4A and 4B are the dose-responses of phosphorylated MEK (pMEK) and double-phosphorylated ERK (ppERK) as a function of PMA concentration, respectively. A smooth positively sloped transfer function is representative of the steady state single cell response to PMA (Fig 4C) and consistent with the widely-accepted fact that MEK phosphorylates ERK. As in the case of the *E. coli* data, we reiterate that neither the dose-responses nor the transfer function contain any signal supporting a causal inference.

Fig 4D is the INDUCE analysis using a two-node network model (compare to Fig 2A and 2D). As predicted by the model, the variance of pMEK hardly depends on Cov(pMEK, ppERK) whereas the variance of ppERK is quadratic in Cov(pMEK, ppERK). Interpreted through the lens of INDUCE analysis, the data is consistent with the causation prediction that MEK phosphorylates ERK. That the regulation is an activation can be inferred from the positive sign of Cov(pMEK, ppERK).

Given that MEK and ERK are at the terminus of a signaling cascade downstream of the effector of PMA ligand (PKC → Ras → Raf → MEK → ERK): should we expect open loop trajectories predicted by the cascade network model in Fig 2I and 2L? The theory and modeling experiments show that open loop trajectories, resulting from the interaction of two or more noise sources (e.g, Ras and MEK), only occur for certain biochemical parameterizations of the cascade network motif (Fig. B in S1 text). The INDUCE analysis shows no sign of an open loop trajectory, therefore we conclude that potential noise sources upstream of MEK exhibit variance-covariance characteristics similar to Fig. B(m-p) in S1 text, which is indistinguishable from an isolated two-node network. Fitting the simpler two node model as a criterion for edge directionality prediction simplifies the analysis for causality prediction and can be used for edges embedded in different network topologies when there is empirical support for the simplification.

### Statistical test for causation

To quantify our statistical confidence in our causation predictions, we compared the fits of two competing causation hypotheses. Model 1 corresponds to the hypothesis that MEK regulate ERK. Alternatively, model 2 assumes that ERK regulates MEK. Let $\epsilon_{i}^{{\text{Model}}_{1}}$ represent the errors in the fit associated with the first model at the *i*-th of the 23 applied PMA doses, that is

$$\begin{array}{ccc} \epsilon_{i}^{{\text{Model}}_{1}} & = & {\left[Var\left(pMEK_{i}^{\text{actual}}\right)-Var\left(pMEK_{i}^{{\text{Model}}_{1}}\right)\right]}^{2}+\\ & & {\left[Var\left(ppERK_{i}^{\text{actual}}\right)-Var\left(ppERK_{i}^{{\text{Model}}_{1}}\right)\right]}^{2}, \end{array}$$ (15)

and let $\epsilon_{i}^{{\text{Model}}_{2}}$ be similarly constructed representing the errors in the fit associated with the second model. If model 1 results in a significantly better fit to the data than model 2, we expect $\epsilon_{i}^{{\text{Model}}_{1}}$ to be consistently smaller than $\epsilon_{i}^{{\text{Model}}_{2}}$. As the data points to be fitted by the two models are the same, this is a paired comparison between Model 1 and Model 2. Therefore we can use the non-parametric Wilcoxon signed rank test to test the null hypothesis that the two models have statistically similar fitting errors. If the null hypothesis is rejected, then we favor the alternative hypothesis that corresponds to the Model which has consistently the smaller errors, and compute its p-value using a one-sided version of the Wilcoxon signed-rank test. The statistical significance of our causation predictions for both the MEK-ERK and the G1-G2 examples presented previously are shown in Table 1. In both cases we reject the null hypothesis with a very small and significant p-value, with the favored alternative hypotheses corresponding to the known correct model of the respective system.

**Table 1:** Statistical significance test for causation.

Inference	*p*-value	N
G1 → G2 (*E. coli*)	1.2 × 10^−7^	23
MEK → ERK (T cell)	1.5 × 10^−5^	24

## Discussion

The insight of the INDUCE analysis is that the covariance matrix undergoes predictable and measurable changes in response to changes in the connectivity strength of the network, and that this phenomenon can be applied to causation prediction. Changing connectivity strength via dose-response is a very mild type of intervention (in the sense of Pearl [2]). Protein concentration changes evoke a natural property of biomolecular networks: connectivity strength is a nonlinear function of protein concentration. Chemical ligands (e.g., drugs) provide a convenient experimental handle for modulating connectivity strength in biochemical networks. A limitation to our analysis is our assumption that the noise affecting different nodes (matrix Q in Eq 3) does not depend on the chemical ligand. While this is an approximation, it may not be true in general. A criterion to rule out this possibility is discussed in Section G of S1 text.

We report the first description of noise propagation using the experimentally measured covariance matrix of single cell phospho-protein in an endogenous mammalian signaling network. More than simply detecting the phenomenon, our method enables inferences about the network neighborhood of the measured species. Convergent activation of ERK by regulators other than MEK was ruled-out by complete absence of ERK activation given pre-treatment with MEK inhibitor. Prior to this analysis it was unknown whether noise propagates over multiple “hops.” Though the intracellular ligand, PMA, stimulates the pathway a few hops upstream of MEK (at the level of PKC), the experimental data showed no evidence of multi-hop noise propagation—there was no discernible complexity beyond the predicted two-node covariance matrix sensitivity plot (compare Figs 3 and 4 to Fig 2A and 2D). (See Section E in S1 text for extended discussion). It may be a robust feature of the MAP kinase pathway that noise does not propagate beyond direct interactions. Alternatively, more physiologically realistic activation (e.g., ligands targeting trans-membrane receptors) might have different noise propagation characteristics.

Empirically, we observed that the concentration distributions of signaling proteins in mammalian cells are better approximated by the lognormal rather than the normal distribution. Because signaling proteins are expressed at thousands of copies per cell, signaling noise can not be attributed to low-copy-number effects as modeled by the chemical Langevin equation. We found it necessary to include stochastic reaction rate constants in our equations as a source of fluctuations to recapitulate the empirical lognormal distributions of protein concentrations. Because of the multiplicative structure of chemical processes, small differences in reaction rate constants result in large deviations in protein concentration from average. In summary, we model the logarithm of protein concentration as the output of a linear stochastic dynamical system driven by additive white noise (Eq 3). Though the model was motivated by empirical lognormal protein concentrations in mammalian cells, it happens to also to be generally applicable to low-copy-number fluctuations in *E. coli* gene expression since the chemical noise terms in the CLE formulation are absorbed into the dominating reaction rate noise terms.

Causation prediction using our method is accessible to experimentalists in the form of an easy-to-implement graphical technique and statistical test. The INDUCE analysis enables causality inference even using destructive assays that preclude the acquisition of time series data.

## Materials and Methods

### Data analysis

To facilitate comparing different dose-response experiments and/or combining data from multiple dose-response experiments in a single analysis, fluorescence values were normalized in the linear scale by the *K*_*ji*_ and maximum amplitude of the response. The logarithm of the normalized fluorescence values were further analyzed. The covariance matrix of the log-fluorescence was estimated at each ligand dose by fitting the bivariate normal distribution. The covariance matrix elements were then used to estimate three noise parameters in the model for an isolated network connection. (See Section F in S1 text for details.)

### Mice, antibodies, and reagents

Splenocytes and lymphocytes were isolated from B10A.CD3*ϵ*-/- mice (Taconic farms) or 5C.C7 TCR transgenic mice (Taconic) on a Rag-2-/- background and used to prepare cultures of primary cells (see below). E10 antibody against ERK[pT202pY204] and 166F8 antibody against MEK1/2[pS221] were purchased from Cell Signaling Technology (Beverly, Massachusetts, United States); 30-F11 antibody against CD45 was from eBioscience (San Diego, California, United States). Secondary antibodies coupled to fluorescent dye were from Jackson ImmunoResearch (West Grove, Pennsylvania, United States). FACS buffer consisted of 10% fetal bovine serum (MSKCC tissue culture core facility) and 0.1% sodium azide in PBS. DAPI was purchased from Dojindo. All cell cultures were prepared in complete medium prepared by the MSKCC core media preparation facility (this medium contained RPMI-1640 augmented with 10% fetal bovine serum + 10μg/ml penicillin-strep + 2mMol glutamine + 10mM HEPES (pH7.0) + 1mMol sodium pyruvate + 0.1mMol non-essential amino acids + 50*μ*Mol *β*-mercaptoethanol. Recombinant mouse IL-2 was obtained from eBioscience (San Diego CA).

The animal protocol was reviewed and approved by the Institutional Animal Care and Use Committee (IACUC) of the Memorial Sloan Kettering Cancer Center (New York NY). The protocol number is 05-12-031 (last renewal data: December 23rd 2013). Mice (older than 4-week old) were exposed to 100% carbon dioxide at 5 PSI for a minimum of 3 minutes in a cage or euthanasia chamber as recommended in RARC’s Euthanasia Guidelines for Investigators. The mice were left undisturbed for an additional 15 minutes. Prior to disposal or tissue collection, death was confirmed by palpating for the absence of an apex heart beat and a lack of respiration.

### Preparation of stimulated primary T lymphocytes

5C.C7 T cell cultures were prepared as follows. B10A.CD3*ϵ*-/- splenocytes were irradiated with 3000rad, washed once, and used as stimulator/feeder cells. 5C.C7 T cells were harvested from axillary, lateral auxiliary and inguinal lymph nodes as well as spleen, and mixed with MCC peptide and B10A.CD3ϵ-/- splenocytes in complete RPMI. After two days, cells were expanded by diluting 2 fold into medium containing 100 pM IL-2. After four days, the cells were again expanded by 2 fold dilution into medium with IL-2. After one more day of culture, cells were harvested and spun through a Ficoll-Paque Plus gradient (GE Healthcare) to remove dead cells. T cells were recovered, washed twice in complete medium and resuspended at 1 million/ml in complete medium with 100pM IL-2. Cells were used for experiments between 6 and 8 days after primary stimulation.

### T cell activation and antibody staining protocol

T cells were activated in their medium of culture in a V-bottom 96-well plate (Corning). Serial dilution of PMA (in a serial dilution of DMSO) was added to the cells. Plates containing T+PMA solutions were placed on a water bath at 37°C and incubated for 10 min. After activation plates were put on ice with 4% ice-cold paraformaldehyde added directly to T+PMA solution for 15 minutes (final working dilution 1.6% paraformaldehyde). Cells were then permeabilized with ice-cold 90% methanol for 15 min on ice, and washed twice with FACS buffer. Cells were then labeled with a combination of anti-ppERK, and anti-pMEK primary antibodies for 30 min at room temperature, followed by a combination of secondary antibodies and anti-CD45 for 30 minutes at room temperature.

### FACS analysis of cellular signaling response

Cells were loaded in FACS buffer with DAPI and their immunofluorescence acquired on a LSRII instrument (BDBioscience). Electronic compensation and pre-data processing was performed to select singlet cells (based on light scattering characteristics) with positive expression of CD45 (a T cell marker) and positive DAPI staining. Further analysis was performed with ad hoc computing tools.

## Supporting Information

S1 text Supporting Information. Consolidated supporting information document.
(PDF)
