# Supplementary material for: Noise-Driven Causal Inference in Biomolecular Networks
Source: PLoS One. 2015 Jun 1;10(6):e0125777. doi: 10.1371/journal.pone.0125777 (PMC4452541; doi:10.1371/journal.pone.0125777)
Supplement: S1 text — Consolidated supporting information document. (PDF) [file pone.0125777.s001.pdf]

**S1 Text: Supporting Information**

# Noise-driven causal inference in biomolecular networks

Robert J. Prill, Robert Vogel, Guillermo A. Cecchi, Grégoire Altan-Bonnet, and Gustavo Stolovitzky

## Contents

**A. Fluctuations of pMEK and ppERK in response to PMA activation** **2**

**B. Derivation of stochastic equations with parameter fluctuations in log-variables** **3**

**C. INDUCE analysis under different topologies** **5**

C.1 Isolated link with global noise term . . . . . . . . . . . . . . . . . . . . . . . . . . . . . . . . 5

C.2 Fan-in network motif . . . . . . . . . . . . . . . . . . . . . . . . . . . . . . . . . . . . . . . . . 6

C.3 Cascade network configuration . . . . . . . . . . . . . . . . . . . . . . . . . . . . . . . . . . . 6

**D. Parameters choices in Figure 2** **7**

**E. Sensitivity analysis of variance-covariance loop formation** **7**

**F. Data Analysis** **9**

**G. Criteria to address the assumption that Q is independent of the stimulus** **9**

## A. Fluctuations of pMEK and ppERK in response to PMA activation

Our analysis approximates signal transduction to a series of logarithmic stochastic chemical reactions. The necessity of the log transformation was developed from our observed phospho-flow cytometry measurements of phorbol 12-myristate 13-acetate (PMA) activated T cells (Fig. A). Visually, these log-scale data exhibit ellipsoidal probability densities, a hallmark of normality. Quantitatively, the superiority of the log-scale can be determined by a statistical test and goodness of fit measure. One such prescription is to compute Wilcoxon signed-rank test comparing the square residuals from each of the normal and log-normal distribution fits, followed by an analysis of the sum-square residuals. Application of this method indicated that the residuals are not equivalent ($p = 2.69$ x $10^{-14}$), and the sum-square residuals showed $\approx 10$ fold preference for the log-normal distribution description. Overall, the necessity of the log transform can be assessed graphically. In situations where a more precise definition is needed a statistical test and a goodness of fit can be used.

Activation of the Mitogen Activated Protein Kinase (MAPK) cascade has been shown to exhibit ultra-sensitivity (steep transfer functions [1]) and feedback regulation [2, 3, 4]. Our data exhibits a steep activation of ppERK by pMEK (ultrasensitive), unimodal, and graded response to PMA (Fig. A). Our data does not exhibit the classic hallmarks of feedback, multistability or a non-monotonic average response to stimuli. We are not the first to measure graded responses of the MAPK pathway to stimulation. The seminal work of Huang and Ferrell demonstrated that a tiered, feedback free, biochemical cascade is sufficient to exhibit ultrasensitive activation of MAPK in *Xenopus* oocyte extracts [2]. In context to single cell measurements of T cells, Prasad *et al.* showed a smooth, unimodal, and monotonic increase of ppERK when activating T cells with PMA. This was remarkably different to their observation of a bimodal response when activating T cells through the T cell receptor (TCR) [4]. These studies and our own, demonstrate that the response of MAPK to stimuli is diverse and context dependent.

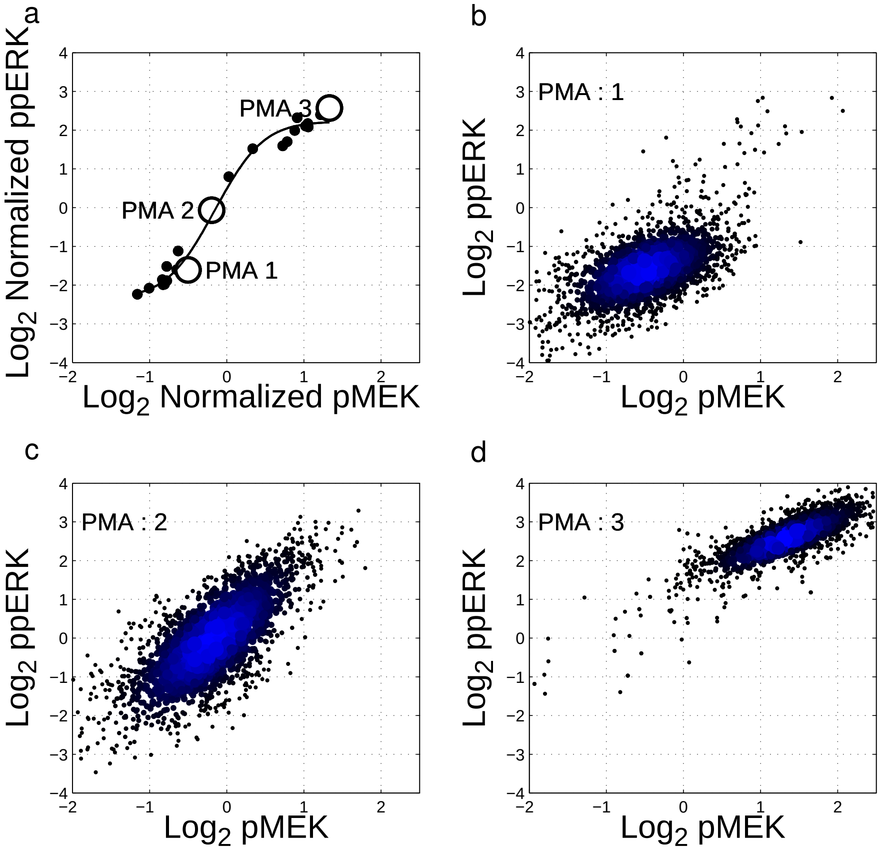


Figure A: (a) The transfer function from pMEK→ppERK, of PMA stimulated T lymphocytes. (b-d) Single cell flow cytometry measurements for the respective PMA dose. The size and shade of blue represents the probability density of cells at that location. These figures demonstrate that pMEK and ppERK are activated unimodally and smoothly, qualitatively maintaining a bivariate log-normal distribution with increasing doses of PMA.

# B. Derivation of stochastic equations with parameter fluctuations in log-variables

Our starting point for modeling biochemical reactions is the chemical Langevin equation

$$\frac{dx_j}{dt} = \sum_{i \neq j} (f_j(x_i;\theta_{ji})) - \lambda_j x_j + \frac{1}{\Omega^{1/2}} \sqrt{\sum_{i \neq j} (f_j(x_i;\theta_{ji})) + \lambda_j x_j}\, \xi_j(t) \tag{S1}$$

where $f_j(x_i;\theta_{ji})$ is the rate of production of chemical species $j$ controlled by chemical species $i$, $\theta_{ji}$ is the set of parameters that determine the function $f_j(x_i;\theta_{ji})$, $\lambda_j$ is the rate constant for the degradation of species $j$, and $\Omega$ is the volume of the cell. The production rate $f_j(x_i;\theta_{ji})$ is a nonlinear, saturating function representing enzymatic activity—e.g., the Hill equation. Writing the parameters as quantities that fluctuate

around their mean values, $\lambda_j = \langle\lambda_j\rangle + \delta\lambda_j$, and $\theta_{ji} = \langle\theta_{ji}\rangle + \delta\theta_{ji}$, we have

$$\begin{aligned}\frac{dx_j}{dt} &= \sum_{i\neq j}\left(f_j(x_i;\langle\theta_{ji}\rangle + \delta\theta_{ji})\right) - (\langle\lambda_j\rangle + \delta\lambda_j)x_j \\ &+ \frac{1}{\Omega^{1/2}}\sqrt{\sum_{i\neq j}\left(f_j(x_i;\langle\theta_{ji}\rangle + \delta\theta_{ji})\right) + (\langle\lambda_j\rangle + \delta\lambda_j)\,x_j}\;\xi_j(t).\end{aligned} \tag{S2}$$

The inclusion of the parameter fluctuations in the square root terms assumes that the intrinsic fluctuations $\xi_j(t)$ are faster than the parameter fluctuations $\delta\theta_{ji}$ and $\delta\lambda_j$. Writing $x_j$ as a quantity that fluctuates around its mean value $(\langle x_j\rangle + \delta x_j)$ we expand Equation S2 using the size of the fluctuations as the order parameter, resulting in terms of order zero (those that do not depend on the fluctuating terms), those that depend on the fluctuating terms to the first order, and those that depend on products of two or more fluctuating terms. Disregarding the latter, we obtain

$$\begin{aligned}\frac{d\langle x_j\rangle}{dt} + \frac{d\delta x_j}{dt} &= \sum_{i\neq j}\left(f_j(\langle x_i\rangle;\langle\theta_{ji}\rangle) + \frac{\partial f_j}{\partial x_i}\delta x_i + \frac{\partial f_j}{\partial\theta_{ji}}\delta\theta_{ji}\right) - (\langle\lambda_j\rangle + \delta\lambda_j)\langle x_j\rangle - \langle\lambda_j\rangle\delta x_j \\ &+ \frac{1}{\Omega^{1/2}}\sqrt{\sum_{i\neq j}\left(f_j(\langle x_i\rangle,\langle\theta_{ji}\rangle)\right) + \langle\lambda_j\rangle\langle x_j\rangle}\;\xi_j(t)\end{aligned} \tag{S3}$$

This equation can be solved hierarchically, order by order. In the stationary state, the zero-th order terms result in

$$\langle x_j\rangle = \frac{1}{\langle\lambda_j\rangle}\sum_{i\neq j} f_j(\langle x_i\rangle;\langle\theta_{ji}\rangle) \tag{S4}$$

The first order terms give the equation for the fluctuations in species $j$

$$\frac{d\delta x_j}{dt} = \sum_{i\neq j}\left(\frac{\partial f_j}{\partial x_i}\delta x_i + \frac{\partial f_j}{\partial\theta_{ji}}\delta\theta_{ji}\right) - \langle\lambda_j\rangle\delta x_j - \delta\lambda_j\langle x_j\rangle + \frac{1}{\Omega^{1/2}}\sqrt{2\langle\lambda_j\rangle\langle x_j\rangle}\;\xi_j(t) \tag{S5}$$

Changing to logarithmic variables, let $y_j = \ln(x_j) = \ln(\langle x_j\rangle + \delta x_j)$. Then to the first order in $\delta x_j$ we have

$$y_j = \ln(\langle x_j\rangle) + \frac{\delta x_j}{\langle x_j\rangle} + \mathcal{O}(\delta x_j^2) \tag{S6}$$

and $\langle y_j\rangle = \ln(\langle x_j\rangle)$ given that $\langle\delta x_j\rangle = 0$. The logarithmic increment in species $j$ is

$$\delta y_j = \ln(x_j) - \ln(\langle x_j\rangle) = \frac{\delta x_j}{\langle x_j\rangle} \tag{S7}$$

Dividing Equation S5 by $\langle x_j\rangle$ we find the equations for the fluctuations in logarithmic scale

$$\frac{d\delta y_j}{dt} = \sum_{i\neq j}\left(\langle\lambda_j\rangle\frac{\partial\ln f_j}{\partial\ln x_i}\delta y_i + \frac{\langle\lambda_j\rangle}{\langle\theta_{ji}\rangle}\frac{\partial\ln f_j}{\partial\ln\theta_{ji}}\delta\theta_{ji}\right) - \langle\lambda_j\rangle\delta y_j - \delta\lambda_j + \sqrt{2\frac{\langle\lambda_j\rangle}{\Omega\langle x_j\rangle}}\;\xi_j(t) \tag{S8}$$

Equation S8 is the chemical Langevin equation with fluctuating parameters in log-scale variables linearized about a fixed point at $\langle x_j\rangle$.

When the abundance of the chemical species represented by $\Omega\langle x_j\rangle$ is large, the fluctuations represented by the last term in Equation S8 are small. Formally this is attained by making the volume $\Omega$ tend to infinity.

Then, the only remaining fluctuations in Equation S8 are the fluctuations in the parameters, which we model as Wiener processes (i.e., fluctuations are fast compared with the half life of the species)

$$\sum_{i \neq j} \left( \frac{\langle \lambda_j \rangle}{\langle \theta_{ji} \rangle} \frac{\partial \ln f_j}{\partial \ln \theta_{ji}} \delta\theta_{ji} \right) - \delta\lambda_j = q_j \epsilon_j + q_G \epsilon_G \tag{S9}$$

where the $\epsilon$'s are delta-correlated white noise processes. Approximating the left hand side by the sum of two random variables with constant coefficients is consistent with our experimental observations (Fig. C). Note that the parameter fluctuations are modeled by one term that is independent for each species ($q_j \epsilon_j$) and one term that is common to all species ($q_G \epsilon_G$) representing a global source of parameter fluctuations. Therefore, we have the linearized system

$$\frac{d\delta y_j}{dt} = \sum_{i \neq j} (a_{ji} \delta y_i) - \langle \lambda_j \rangle \delta y_j + q_j \epsilon_j + q_G \epsilon_G \tag{S10}$$

where $a_{ji} = \langle \lambda_j \rangle \frac{\partial \ln f_j(\langle x_i \rangle, \langle \theta_{ji} \rangle)}{\partial \ln \langle x_i \rangle}$. The general form of these equations for arbitrary regulatory topologies **A** and normally distributed noise models **Q** is

$$\frac{d}{dt} \begin{bmatrix} \delta y_1(t) \\ \vdots \\ \delta y_N(t) \end{bmatrix} = \mathbf{A} \begin{bmatrix} \delta y_1(t) \\ \vdots \\ \delta y_N(t) \end{bmatrix} + \mathbf{Q} \begin{bmatrix} \epsilon_1(t) \\ \vdots \\ \epsilon_N(t) \\ \epsilon_G(t) \end{bmatrix} \tag{S11}$$

where $\delta y_i$ is the logarithmic increment in species $i$, the entries in the matrix **A** are $a_{ji}$ as described above for $i \neq j$ and $a_{jj} = -\lambda_j$, and the entries of **Q** are independent of the log-concentrations.

In summary, log-scale fluctuations in the concentrations of reacting chemical species of sufficiently high concentration follow Equation S11 therefore the analysis presented in the main text applies to fluctuations in log-concentration of proteins.

# C. INDUCE analysis under different topologies

## C.1 Isolated link with global noise term

Model fits to experimental data were produced using a model of the form of

$$\frac{d}{dt} \begin{bmatrix} \delta x_1 \\ \delta x_2 \end{bmatrix} = \begin{bmatrix} -\lambda & 0 \\ k & -\lambda \end{bmatrix} \begin{bmatrix} \delta x_1 \\ \delta x_2 \end{bmatrix} + \begin{bmatrix} q_1 & 0 & q_g \\ 0 & q_2 & q_g \end{bmatrix} \begin{bmatrix} \xi_1 \\ \xi_2 \\ \xi_g \end{bmatrix} \tag{S12}$$

where $k$ is a synthesis rate constant and -$\lambda_1 = -\lambda_2 = -\lambda$ is a degradation rate constant. The simplification of equal degradation rate constants modeling dephosphorylation of the protein species simplifies the calculations and improves intuition. Each variable $\delta x_i$ experiences an intrinsic noise $\xi_i$ and an extrinsic (global) noise $\xi_g$, the magnitude of which is modulated by the coefficients $q_i$ and $q_g$, respectively. Solving Equation 8 for this model gives the covariance matrix

$$\mathbf{\Sigma}_o = \begin{bmatrix} \eta_g + \alpha & \sigma_{12} \\ \sigma_{12} & \beta + \frac{\alpha\eta_g + \eta_g^2 - 2\eta_g\sigma_{12} + 2\sigma_{12}^2}{\eta_g + \alpha} \end{bmatrix} \tag{S13}$$

where $\alpha = \frac{q_1^2}{2\lambda}$ and $\beta = \frac{q_2^2}{2\lambda}$ are components of the variances that do not depend on the network connectivity, and $\eta_g = \frac{q_g^2}{2\lambda}$ is the global noise term. When the global noise $\eta_g \to 0$ Equation S13 is equivalent to Equation 12 in the main text.

## C.2 Fan-in network motif

Here we expand upon the presentation of the model equations in the **Materials and Methods**. In the fan-in network motif, protein 2 is regulated by proteins 1 and 3.

$$\frac{d}{dt}\begin{bmatrix}\delta x_1\\ \delta x_2\\ \delta x_3\end{bmatrix} = \begin{bmatrix}-\lambda & 0 & 0\\ k_{21} & -\lambda & k_{23}\\ 0 & 0 & -\lambda\end{bmatrix}\begin{bmatrix}\delta x_1\\ \delta x_2\\ \delta x_3\end{bmatrix} + \begin{bmatrix}q_1 & 0 & 0\\ 0 & q_2 & 0\\ 0 & 0 & q_3\end{bmatrix}\begin{bmatrix}\xi_1\\ \xi_2\\ \xi_3\end{bmatrix}. \tag{S14}$$

Equal degradation rate constants modeling dephosphorylation of the protein species simplifies the calculations and improves intuition. Solving Equation 8 for this model gives the covariance matrix entries

$$\sigma_1^2 = \alpha \tag{S15a}$$

$$\sigma_{12} = \frac{\alpha k_{21}}{2\lambda} \tag{S15b}$$

$$\sigma_2^2 = \beta + \frac{\alpha k_{21}^2}{2\lambda^2} + \frac{\gamma k_{23}^2}{2\lambda^2} \tag{S15c}$$

$$\sigma_{13} = 0 \tag{S15d}$$

$$\sigma_{23} = \frac{\gamma k_{23}}{2\lambda} \tag{S15e}$$

$$\sigma_3^2 = \gamma \tag{S15f}$$

where $\alpha = \frac{q_1^2}{2\lambda}, \beta = \frac{q_2^2}{2\lambda}, \gamma = \frac{q_3^2}{2\lambda}$.

## C.3 Cascade network configuration

Here we expand upon the presentation of the model equations in the **Materials and Methods**. In the cascade network motif, protein 3 regulates protein 1, and protein 1 regulates protein 2.

$$\frac{d}{dt}\begin{bmatrix}\delta x_1\\ \delta x_2\\ \delta x_3\end{bmatrix} = \begin{bmatrix}-\lambda & 0 & k_{13}\\ k_{21} & -\lambda & 0\\ 0 & 0 & -\lambda\end{bmatrix}\begin{bmatrix}\delta x_1\\ \delta x_2\\ \delta x_3\end{bmatrix} + \begin{bmatrix}q_1 & 0 & 0\\ 0 & q_2 & 0\\ 0 & 0 & q_3\end{bmatrix}\begin{bmatrix}\xi_1\\ \xi_2\\ \xi_3\end{bmatrix} \tag{S16}$$

Equal degradation rate constants modeling dephosphorylation of the protein species simplifies the calculations and improves intuition. Solving Equation 8 for this model gives the covariance matrix entries

$$\sigma_1^2 = \alpha + \frac{\gamma k_{13}^2}{2\lambda^2} \tag{S17a}$$

$$\sigma_{12} = \frac{\alpha k_{21}}{2\lambda} + \frac{3\gamma k_{21} k_{13}^2}{8\lambda^3}$$

$$\sigma_2^2 = \beta + \frac{\alpha k_{21}^2}{2\lambda^2} + \frac{3\gamma k_{21}^2 k_{13}^2}{8\lambda^4}$$

$$\sigma_{13} = \frac{\gamma k_{13}}{2\lambda} \tag{S17b}$$

$$\sigma_{23} = \frac{\gamma k_{21} k_{13}}{4\lambda^2} \tag{S17c}$$

$$\sigma_3^2 = \gamma \tag{S17d}$$

where $\alpha = \frac{q_1^2}{2\lambda}, \beta = \frac{q_2^2}{2\lambda}, \gamma = \frac{q_3^2}{2\lambda}$.

## D. Parameters choices in Figure 2

Fig. 2 in the main text explores three network connectivities: isolated link, fan-in, and cascade. For the isolated link, the transfer function in logarithmic variables is given by the Hill equation with parameters $\nu$=10, $K$=3, $n$=4, $\lambda$=0.8. The reaction rate $k$ in Equation 11, which we rename $k_{21}$ here, is the instantaneous slope of the transfer function in logarithmic variables. The effect of the parameter $k_{21}$ is explored in the parametric plots of the entries of the covariance matrix governed by Equation 11 with parameters $\alpha = \beta = 1$.

For the fan-in connectivity in which species 1 and 3 regulate species 2, there are two transfer functions giving $k_{21}$ and $k_{23}$. We model the overall activation of node 2 as the sum of in its inputs. The transfer function giving $k_{21}$ is parameterized as above. The transfer function giving $k_{23}$ is parameterized by $\nu$=10, $K$=2.5, $n$=4, $\lambda$=0.8. The equations for variance and covariance are as follows, with $\alpha = \beta = \gamma = 1$.

$$\begin{aligned}\sigma_1^2 &= \alpha \\ \sigma_{12} &= \frac{\alpha k_{21}}{2\lambda} \\ \sigma_2^2 &= \beta + \frac{\alpha k_{21}^2}{2\lambda^2} + \frac{\gamma k_{23}^2}{2\lambda^2},\end{aligned}$$

For the cascade connectivity in which species 3 regulates 1 and species 1 regulates 2, there are two transfer functions giving $k_{13}$ and $k_{21}$. The transfer function giving $k_{21}$ is parameterized by $\nu$=10, $K$=6.5, $n$=4, $\lambda$=0.7, and the transfer function giving $k_{13}$ is parameterized by $\nu$=10, $K$=3, $n$=3, $\lambda$=0.7. The equations for variance and covariance are as follows, with $\alpha = \beta = \gamma = 1$.

$$\begin{aligned}\sigma_1^2 &= \alpha + \frac{\gamma k_{13}^2}{2\lambda^2} \\ \sigma_{12} &= \frac{\alpha k_{21}}{2\lambda} + \frac{3\gamma k_{21} k_{13}^2}{8\lambda^3} \\ \sigma_2^2 &= \beta + \frac{\alpha k_{21}^2}{2\lambda^2} + \frac{3\gamma k_{21}^2 k_{13}^2}{8\lambda^4}\end{aligned}$$

## E. Sensitivity analysis of variance-covariance loop formation

In a linear cascade the effect of an upstream node on variance/covariance loop formation depends on the biochemical parameters. The degree of "openness" of the variance/covariance loop is reflective of disparate sensitivities between interacting components. Fig. B illustrates that the loop is closed (and therefore undetectable in real data) when, for example, the sensitivity of measured pathway components are similar. In general, if loops are not observed in real data, one can assume that the link is isolated for the purpose of causality inference which simplifies the analysis and suggests a prescription for applying to a large network one link at a time. The measured activation of MAPK pathway in T cells follows this trend; the sensitivities of cells to PMA stimulation are near identical and the variance/covariance plots do not exhibit the complex looping feature.

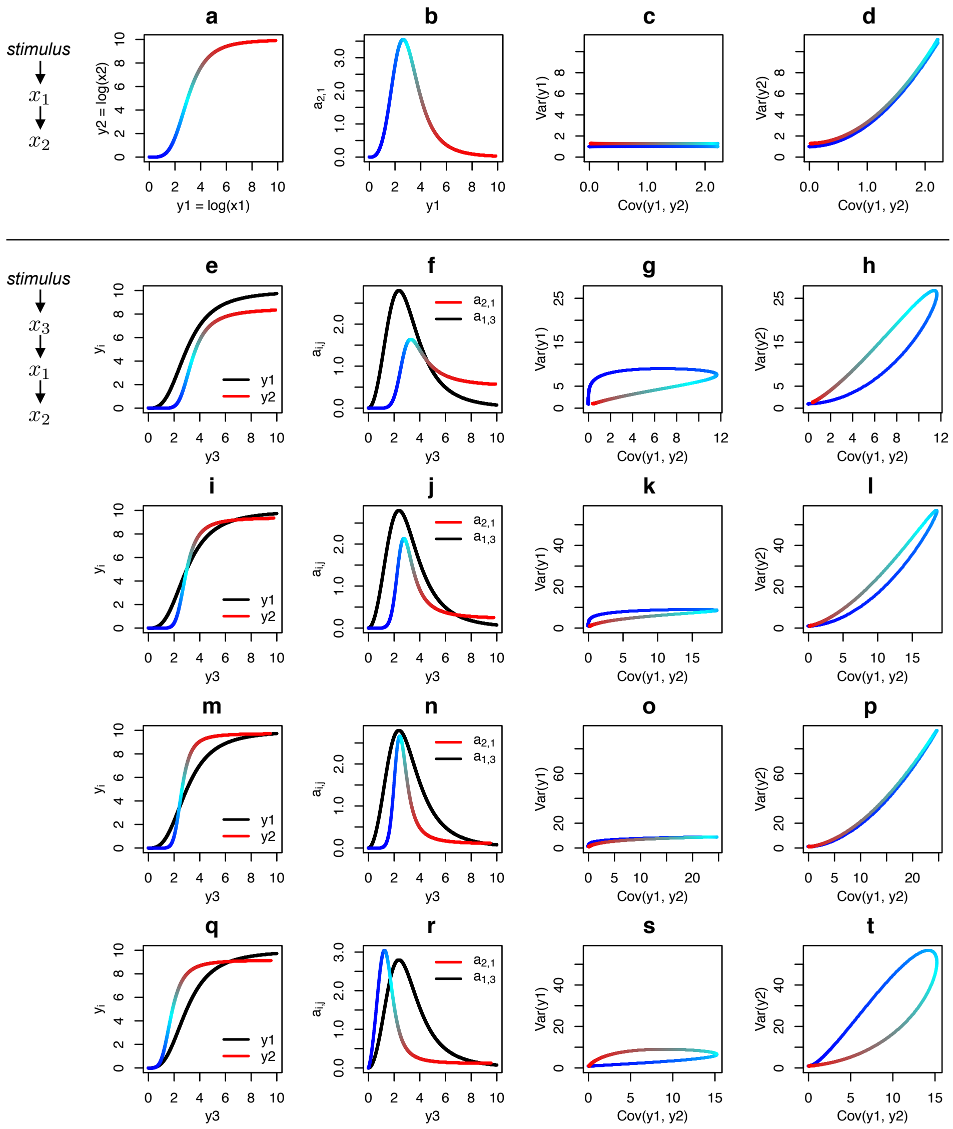

Figure B: **Sensitivity analysis of V-C loop formation.** (a-d) Analysis of a single isolated network link is reprinted from Fig. 2 in the main text. From left to right, (a) the Hill equation model of log concentration is color registered to $y_2$. Compare this series of panels to (m-p). (b) The derivative of the transfer function is the network connection strength $a_{21}$, which peaks at half-maximal activation. (c-d) Variance versus covariance shows no loops. (e-h) Analysis of a linear cascade reprinted from Fig. 2 in the main text. Variance versus covariance plots have loops. (i-l) For the same connectivity and different biochemical parameters, loops in the variance versus covariance plots become more "closed." (m-n) Biochemical parameters demonstrating complete closure of variance versus covariance loops. (n) The connection strengths of the two transfer functions peak at the same concentration of $y_3$, the condition for loop closure. (q-r) A biochemical parameterization demonstrating a reversal of loop directions relative to the previous examples. (r) Note that the relative ordering of the connection strength peaks with respect to $y_3$ are reversed from previous examples.

## F. Data Analysis

To facilitate comparing different dose-response experiments and/or combining data from multiple dose-response experiments in a single analysis, fluorescence values were normalized in the linear scale by the half-maximum activation constant, $K$ and maximum amplitude of the response, $A$, using a Hill function.

$$f = A\frac{x^n}{x^n + K^n} + b \tag{S18}$$

The historical *E. coli* dataset consisted of two replicates of the IPTG dose-response which we normalized and combined for our analysis. For Gene 1 the Hill coefficient ($n$) was constrained to be positive and for Gene 2 constrained to be negative. The data amplitude was then normalized by parameter $A$ and the concentration of IPTG was normalized by $K$ of Gene 1. Normalized data from two data sets were then combined such that Gene 1 and Gene 2 expression are functions of the rescaled IPTG concentrations (Fig. 3 in main text).

We applied the same procedure to normalize the T-cell dose-response to PMA using positive Hill coefficients. It was not necessary to combine multiple T-cell dose-response experiments because we finely sampled the dose response curve using small increments of PMA concentration.

Following normalization all subsequent analysis was performed on the $\log_2$ florescence values for the *E. coli* and T-cell experiments. The entries of the covariance matrix were estimated at each dose using curve-fitting by least-squares. We fit the bivariate normal distribution to the log-florescence values. Noise parameters, $\alpha$, $\beta$, and $\eta_g$ were estimated by the least-squares fit of the following equation.

$$\mathbf{\Sigma}_o = \begin{bmatrix} \eta_g + \alpha & \sigma_{12} \\ \sigma_{12} & \beta + \frac{\alpha\eta_g + \eta_g^2 - 2\eta_g\sigma_{12} + 2\sigma_{12}^2}{\eta_g + \alpha} \end{bmatrix}. \tag{S19}$$

When the global noise $\eta_g \to 0$ Equation S19 is equivalent to Equation 12 in the main text.

## G. Criteria to address the assumption that Q is independent of the stimulus

In our derivations we assumed that the elements of the noise strength matrix **Q** were constant with respect to the strength of the stimulus. To support this assumption in the context of the data analyzed in this paper, we studied the dependence of the noise coefficient $\beta$ (proportional to the square of the matrix element $q_2$ representing the intrinsic noise of ppERK, see Eq. 13) as a function of the phosphorylation level of ERK. From the expression for $\sigma_2^2$ in Eq. 13 we can see that the variance of ppERK includes both the intrinsic noise $\beta$, the global noise $\eta_g$ and the noise propagated to ppERK from pMEK. Fig. C shows that the dependence of $\beta$ on ppERK is indeed very mild. The range of the variation of $\beta$ over all values of ppERK is 5 times smaller than the changes of the overall variance of ppERK. Therefore most of the variation in the variance of ppERK is due to the fluctuations in ppERK propagated from pMEK.

In order to decide if INDUCE is applicable in specific situations we next present a condition that is necessary for the constant **Q** requirement. We will use the case of an isolated link with intrinsic and global noise terms which was discussed in Section 3.1. In particular we will focus on the noise terms $\beta + \eta_g$ representing a combination of the intrinsic noise in ppERK and the global noise. From Eq. 13 the variance of ppERK can be expressed as

$$\sigma_2^2 = \beta + \eta_g\left(1 - 2\frac{\sigma_{12}}{\sigma_1^2}\right) - 2\frac{\sigma_{12}^2}{\sigma_1^2}. \tag{S20}$$

Let's now consider two extreme conditions: one in which the stimulus $s$ is the lowest ($s_0 = 0$) and the other in which the stimulus is maximum ($s_{max}$). It is usually the case that these two extremes correspond to

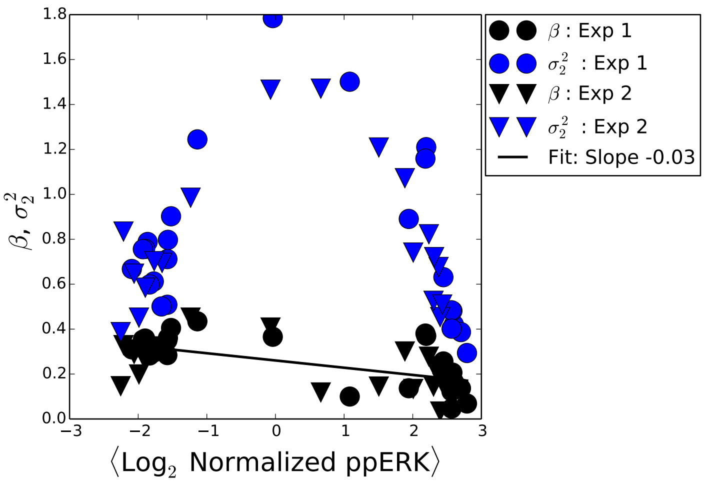


Figure C: The noise coefficient $\beta$ (proportional to the square of the matrix element $q_2$ representing the intrinsic noise of ppERK, see Eq S13) plotted as a function of the phosphorylation level of ERK. $\beta$ is estimated by linear regression of rearranged Eq. S20 (correlation coefficient 0.77). A linear fit of $\beta$ as a function of phosphorylated ERK levels exhibits a slope of -0.03 and therefore the noise is only mildly dependent on the signal strength. The blue symbols are the variance of the total fluctuations of ppERK that includes the noise acting directly on ERK and the fluctuations propagated from MEK.

inactive and saturated responses of the biochemical reaction under study, and in this sense one should expect $\sigma_{12} \approx 0$. In this case

$$\sigma_2^2(s_{max}) - \sigma_2^2(s_0) = [\beta(s_{max}) + \eta_g(s_{max})] - [\beta(s_0) + \eta_g(s_0)] \tag{S21}$$

If $\beta$ and $\eta_g$ were constant, the above equation should be approximately equal to 0. Indeed this is what we observe in Figs. 3D and 4D, where for the minimum and maximum values of the stimulus, $\sigma_2^2$ coalesce and the covariance attains the smallest absolute values. Fig. D illustrates this effect for the isolated two node model in which $y_1 \to y_2$. The color in the figure represents the strength of the stimulus, with blue and red indicating low and high stimulus respectively. The transfer function of the reaction is shown in Fig. Da. If the coefficients in the noise matrix are constant, as the theory assumes, we should observe a variance-covariance plot of the type shown in Fig. Db. If the noise coefficients vary with the stimulus, for example the noise increases with the stimulus, the variance covariance plot would look like Fig. Dc, for which the values of var($y_2$) at the minimum and maximum stimuli are clearly different.

To make this criterion more quantitative, we have to compare the difference in the $\beta$ and $\eta_g$ coefficients shown in Eq. 21 to the overall range of variation $\sigma_2^2$ across all the stimuli, of which $\beta$ and $\eta_g$ are components. The use of INDUCE would be justified if the variations in $\beta + \eta_g$ are only a small component of the overall variation in $\sigma_2^2$. Let's denote $\sigma_{2,max}^2$ (usually realized at intermediate levels of stimulation) and $\sigma_{2,min}^2$ the maximum and minimum values of $\sigma_2^2$ across all experiments. If $\beta + \eta_g$ is approximately constant, we should have

$$\frac{\Delta[\beta + \eta_g]}{\Delta\sigma_2^2} \equiv \frac{\sigma_2^2(s_{max}) - \sigma_2^2(s_0)}{\sigma_{2,max}^2 - \sigma_{2,min}^2} \ll 1 \tag{S22}$$

Therefore we recommend as a criterion to not use INDUCE if $\frac{\Delta[\beta+\eta_g]}{\Delta\sigma_2^2}$ is bigger than, say, 0.3.

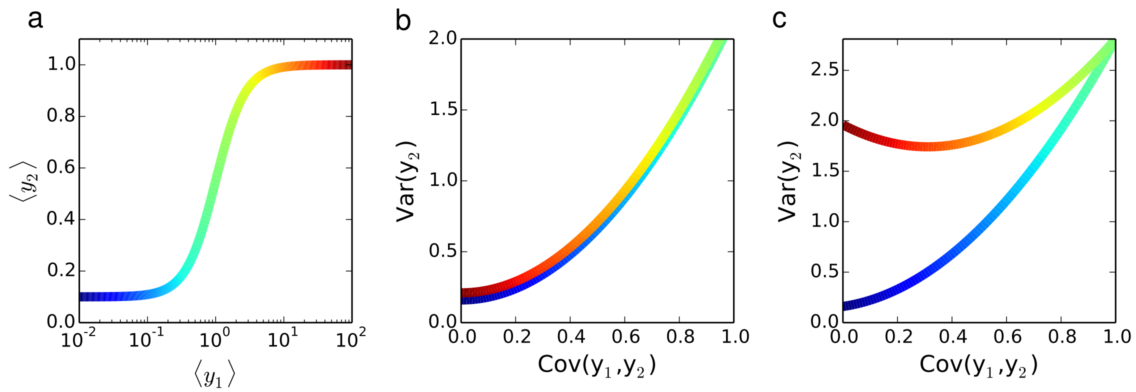


Figure D: Transfer function (a), variance-covariance plot for stimulus-independent noise coefficients (b) and variance-covariance plot for stimulus-dependent (monotonically growing) noise coefficients (c) for the isolated two node model. The color code in the curves represent the strength of the stimulus, with blue indicating low and red high stimulation. For small (in absolute value) covariance values, the behavior of the curves for stimulus-independent coefficients (b) is quite different from the case of stimulus-dependent coefficients (c).

# References


[1] Goldbeter A, Koshland DE. Ultrasensitivity in biochemical systems controlled by covalent modification. Interplay between zero-order and multistep effects. Journal of Biological Chemistry. 1984;259(23):14441–14447.

[2] Huang CY, Ferrell JE. Ultrasensitivity in the mitogen-activated protein kinase cascade. Proceedings of the National Academy of Sciences. 1996;93(19):10078–10083.

[3] Das J, Ho M, Zikherman J, Govern C, Yang M, Weiss A, et al. Digital signaling and hysteresis characterize RAS activation in lymphoid cells. Cell. 2009 Jan;136(2):337–351.

[4] Prasad A, Zikherman J, Das J, Roose JP, Weiss A, Chakraborty AK. Origin of the sharp boundary that discriminates positive and negative selection of thymocytes. Proc Natl Acad Sci U S A. 2009 Jan;106(2):528–533.
